# Editorial

**DOI:** 10.1002/1097-0061(20000930)17:3<169::AID-YEA33>3.0.CO;2-U

**Published:** 2000


### Editorial

There have been two major advances in genomics since the appearance of the last issue of *CFG*. However, these two milestones are of very different character. The first was the joint announcement from the Wellcome Trust and the NIH, in one camp, and Celera Corporation, in the other, of the completion of the 'draft' sequence of the human genome. While there has certainly been an enormous increase in the volume of human genome sequence available to researchers in both the public and private sectors, it is a little difficult to get a grip on just what a 'draft' sequence represents. At the time of writing, estimates of the number of protein-encoding genes in the *Homo sapiens* genome cover a four-fold range and you can even place your bets on the correct number – see the web-site: **http://www.ensembl.org/genesweep.html.** This issue of *CFG*, not wanting to miss out on the fun – to say nothing of adding a considered argument to an important scientific debate – publishes a Review by Ian Dunham (Sanger Centre) that discusses the gene number conundrum and provides a hot tip for those of you that enjoy a flutter.

The second milestone came not from one of the major sequencing centres but from a consortium of labs in the state of Sao Paulo, Brazil. These labs embarked on a genome sequencing project using the 'cottage industry' approach that European researchers had employed in the sequencing of the yeast and *Bacillus* genomes. Like those European efforts, this network approach has proved enormously successful in Brazil, not only producing a complete bacterial genome sequence but also encouraging cooperation between laboratories and raising the general level of technical competence in Sao Paulo state. The genome sequenced is an important one, not only for Sao Paulo but also for science. It is the genome of the plant pathogenic bacterium, *Xylella fastidiosa*. This tick-borne pathogen is causing devastation in the orange groves of Sao Paulo and a close relative is proving a major problem for wine-growers in the USA. This is the first plant pathogen to have its genome sequence determined and the results (in what is probably, at the time of publication, one of the best annotated genomes yet seen; Simpson *et al*., 2000) are of considerable scientific interest. What emerges is that there may be a basic set of genes associated with virulence that are found in both plant and animal pathogens; the *Xylella* sequence, for instance, contains reading-frames specifying haemolysin homologues that had not previously been seen outside of the animal pathogens. Mike Daniels (John Innes Centre) will set this work in context in the next issue of *CFG*. Meanwhile, it is appropriate that we now welcome another researcher from the John Innes Centre to the Board of *CFG*. Mike Gale comes on board with this issue to be Section Editor for Cereal Genomics. With the rice genome sequence well under way and more plant pathogen genomes to come from Sao Paulo, this looks set to be a major area for the Journal.

**S. G. Oliver**
Editor-in-Chief

### References

Simpson AJG, Reinach FC, Arruda P, *et al*. 2000. The genome sequence of the plant pathogen *Xylella fastidiosa*. *Nature* **406**: 151–158.

Copyright © 2000 John Wiley & Sons, Ltd.

## References

[PDF_00067] Simpson A. J., Reinach F. C., Arruda P., Abreu F. A., Acencio M., Alvarenga R., Alves L. M., Araya J. E., Baia G. S., Baptista C. S. (2000). The genome sequence of the plant pathogen Xylella fastidiosa. The Xylella fastidiosa Consortium of the Organization for Nucleotide Sequencing and Analysis.. Nature.

